# Bibliography

**Published:** 1917-12

**Authors:** 


### BIBLIOGRAPHY

ORAL ROENTGENOLOGY. This is a new atlas of X-ray illustrations of remarkable clearness that even the novice can study with great advantage in the cultivation of the faculty of interpreting these pictures from the customary X-ray laboratory. The book is elegantly printed on paper that shows up the cuts to the best advantage. The text is by that careful writer and experimental investigator, Dr. Kurt H. Thoma, author of two other excellent books, "Oral Anesthesia" and "Oral Abscesses." The book is printed by Ritter & Co., of Boston, Mass. This is the best book on this subject we have seen, especially on the dental phase of the subject. We are sure any one who is engaged in X-ray work will read this book, and every general practitioner who is attempting to interpret these pictures will be very much more intelligent by having this work close at hand.

